# Second-line therapy for patients with steroid-refractory aGVHD: systematic review and meta-analysis of randomized controlled trials

**DOI:** 10.3389/fimmu.2023.1211171

**Published:** 2023-06-20

**Authors:** Chengxin Luo, Xiangtao Huang, Ling Wei, Guixian Wu, Yarui Huang, Yaqun Ding, Zhen Huang, Jieping Chen, Xi Li, Yunding Zou, Shuangnian Xu

**Affiliations:** ^1^ Center for Hematology, Southwest Hospital, Third Military Medical University, Chongqing, China; ^2^ Chongqing Key Laboratory of Tumor Immunotherapy, Chongqing Science & Technology Commission, Chongqing, China; ^3^ Department of Hematology, Daping Hospital, Third Military Medical University, Chongqing, China

**Keywords:** acute graft-versus-host disease, steroids-refractory, second-line therapy, mesenchymal stroma cells, ruxolitinib, meta-analysis, randomized controlled trials

## Abstract

**Objective:**

Steroids-refractory (SR) acute graft-versus-host disease (aGVHD) is a life-threatening condition in patients undergoing allogeneic hematopoietic stem cell transplantation (allo-HSCT), but the optimal second-line therapy still has not been established. We aimed to perform a systematic review and meta-analysis of randomized controlled trials (RCTs) to compare the efficacy and safety of different second-line therapy regimens.

**Methods:**

Literature search in MEDLINE, Embase, Cochrane Library and China Biology Medicine databases were performed to retrieve RCTs comparing the efficacy and safety of different therapy regimens for patients with SR aGVHD. Meta-analysis was conducted with Review Manager version 5.3. The primary outcome is the overall response rate (ORR) at day 28. Pooled relative risk (RR) and 95% confidence interval (CI) were calculated with the Mantel-Haenszel method.

**Results:**

Eight eligible RCTs were included, involving 1127 patients with SR aGVHD and a broad range of second-line therapy regimens. Meta-analysis of 3 trials investigating the effects of adding mesenchymal stroma cells (MSCs) to other second-line therapy regimens suggested that the addition of MSCs is associated with significantly improvement in ORR at day 28 (RR = 1.15, 95% CI = 1.01–1.32, *P* = 0.04), especially in patients with severe (grade III–IV or grade C–D) aGVHD (RR = 1.26, 95% CI = 1.04–1.52, *P* = 0.02) and patients with multiorgan involved (RR = 1.27, 95% CI = 1.05–1.55, *P* = 0.01). No significant difference was observed betwwen the MSCs group and control group in consideration of overall survival and serious adverse events. Treatment outcomes of the other trials were comprehensively reviewed, ruxolitinib showed significantly higher ORR and complete response rate at day 28, higher durable overall response at day 56 and longer failure-free survival in comparison with other regimens; inolimomab shows similar 1-year therapy success rate but superior long-term overall survial in comparison with anti-thymocyte globulin, other comparisons did not show significant differences in efficacy.

**Conclusions:**

Adding MSCs to other second-line therapy regimens is associated with significantly improved ORR, ruxolitinib showed significantly better efficacy outcomes in comparison with other regimens in patients with SR aGVHD. Further well-designed RCTs and integrated studies are required to determine the optimal treatment.

**Systematic review registration:**

https://www.crd.york.ac.uk/PROSPERO/, identifier CRD42022342487.

## Introduction

Allogeneic hematopoietic stem cell transplantation (allo-HSCT) is a key treatment strategy for patients with high-risk hematological malignancies and severe non-malignant hematological disorders. The applications of allo-HSCT continuously increased over the past several decades (1). Despite substantial advancements in transplantation technologies, acute graft-versus-host disease (aGVHD) remains one of the most common complications and crucial contributing factor for transplant-related mortality (TRM) in patients undergoing allo-HSCT (2). Approximately 40%–60% of patients developed moderate to severe (grade II–IV) aGVHD after allo-HSCT despite standard prophylaxis (3, 4). Severe aGVHD is a life-threatening condition, associating with a dismal long-term overall survival (OS) lower than 30% for grade III disease and lower than 5% in patients with grade IV disease (5, 6). Above all, aGVHD is a major challenge in clinical practice limiting the application of allo-HSCT and compromising its benefits.

Steroids remains the first-line therapy for aGVHD, however, only 40%–60% of patients can achieve durable response with initial therapy (7–9). Patients with grade III-IV aGVHD, hyperacute GVHD, older age or multiple organs involvement are associated with high risk of treatment failure (10–12). The prognosis of patients with steroids-refractory aGVHD is extremely poor, with a GVHD-related mortality around 70% at 2 years (10, 13). Currently available second-line therapy for patients with steroids-refractory aGVHD mainly include mycophenolate mofetil (MMF), tacrolimus, anti-thymocyte globulin (ATG), Janus kinase (JAK) 1/2 inhibitor ruxolitinib, interleukin 2 receptor (IL-2R) antibodies (daclizumab, inolimomab, basiliximab), mammalian target of rapamycin (mTOR) inhibitors (sirolimus, everolimus), tumor necrosis factor-alpha (TNF-α) inhibitors (infliximab, etanercept), anti-CD52 antibody alemtuzumab, anti-α4β7 integrin antibody vedolizumab, extracorporal photopheresis (ECP) and mesenchymal stroma cells (MSCs) used alone or in combination (2, 9, 14). However, the optimal second-line therapy still has not been established (2, 15). In this study, we performed a systematic review and meta-analysis of randomized controlled trials (RCTs) comparing different second-line therapies in patients with steroids-refractory aGVHD following allo-HSCT, aiming to provide evidences for regimens selection in clinical practice and clues for future study design.

## Methods

### Literature search and study selection

This study was reported according to preferred reporting items for systematic reviews and meta-analyses (PRISMA) statement (16). The protocol was registered on the International Prospective Register of Systematic Reviews (PROSPERO) and available online (registration number CRD42022342487). We searched MEDLINE (by Ovid), Embase, Cochrane Library and China Biology Medicine (CBM) databases on April 6, 2022 with no date and language restriction, the search procedure was repeated on April 13, 2023. The main search terms are “graft versus host disease” or “GVHD” in combination with “refractory”, “resistant”, “persistent”, “second-line” or “salvage”. Reference lists of eligible trials and relevant reviews were manually checked for additional trials.

Two investigators (CXL and XTH) independently assessed eligibility of retrieved citations; disagreements were resolved by discussion with a third investigator (SNX). The inclusion criteria are: (і) included patients receiving allo-HSCT as therapy for hematological disorders, and developing steroids-refractory aGVHD of any grade following allo-HSCT; (ii) compared the efficacy and safety of two or more different second-line therapy regimens for steroids-refractory aGVHD; (iii) study design is randomized controlled trial (RCT). According to previously published agreements, steroids-refractory aGVHD is defined as: disease progression after at least 3 days of treatment with methylprednisolone ≥ 2 mg/kg/day or equivalent; lack of response after at least 7 days of treatment; or failure to taper the methylprednisolone dose to < 0.5 mg/kg/day or the prednisone dose to < 0.6 mg/kg/day (17, 18). Retrospective studies, single-arm studies, dose-escalating studies, case reports or case series, and *post-hoc* analysis were excluded.

### Data extraction and quality assessment

Two investigators (CXL and LW) independently extracted data on trial characteristics (first author, publication year, study design), patients’ characteristics (age, gender, underlying disease, donor type, graft source, grade of aGVHD), type and dosage of second-line therapy regimens, median follow-up and treatment outcomes. The primary outcome is the overall response rate (ORR) at day 28, which is defined as the proportion of patients who achieved complete response (CR) or partial response (PR) at day 28. Secondary outcomes include CR rate, OS, and adverse events (AEs). For dichotomous data, number of patients with events and total number of patients analyzed were extracted. For time-to-event data, hazard ratios (HRs) and 95% confidence intervals (CIs) were extracted. The ln(HR) and standard error (SE) were calculated directly, or estimated from the log-rank *P* value and the number of events with the previously established methods when the HRs and 95% CIs were not reported (19). For trials with multiple publications, survival data were extracted from the report with the longest follow-up. Predesigned forms were used to extracted data and cross-checked to reach a consensus between the two investigators.

Methodological quality of included RCTs were assessed with the Cochrane risk of bias tool based on following aspects: random sequence generation, allocation concealment, blinding of participants and personnel, blinding of outcome assessment, incomplete outcome data, selective reporting and other bias (20). Two investigators independently performed risk of bias assessment; any disagreements were resolved by consensus.

### Statistical analyses

All statistical analyses were performed with Review Manager version 5.3 (Revman, The Cochrane Collaboration, 2014). For dichotomous data, the pooled risk ratios (RRs) and 95% confidence intervals (CIs) were calculated using the Mantel-Haenszel method. For time-to-event data, the pooled HRs and 95% CIs were calculated using the generic inverse-variance method. Heterogeneity was assessed with Chi-square test and *I^2^* statistic. Random-effects model was used when there is significant heterogeneity (*P* ≤ 0.1, *I^2^* > 50%), otherwise fixed-effects model was used. Subgroup analyses were performed according to the patients’ age (< 18 years and ≥ 18 years), grade of aGVHD, involved organ (skin, gastrointestinal, and liver), and the number of involved organs (single organ and multiorgan). All *P* values are two-sided, and a *P* value of < 0.05 indicated statistical significance except that of heterogeneity test.

## Results

### Characteristics of included trials

**Figure 1:**
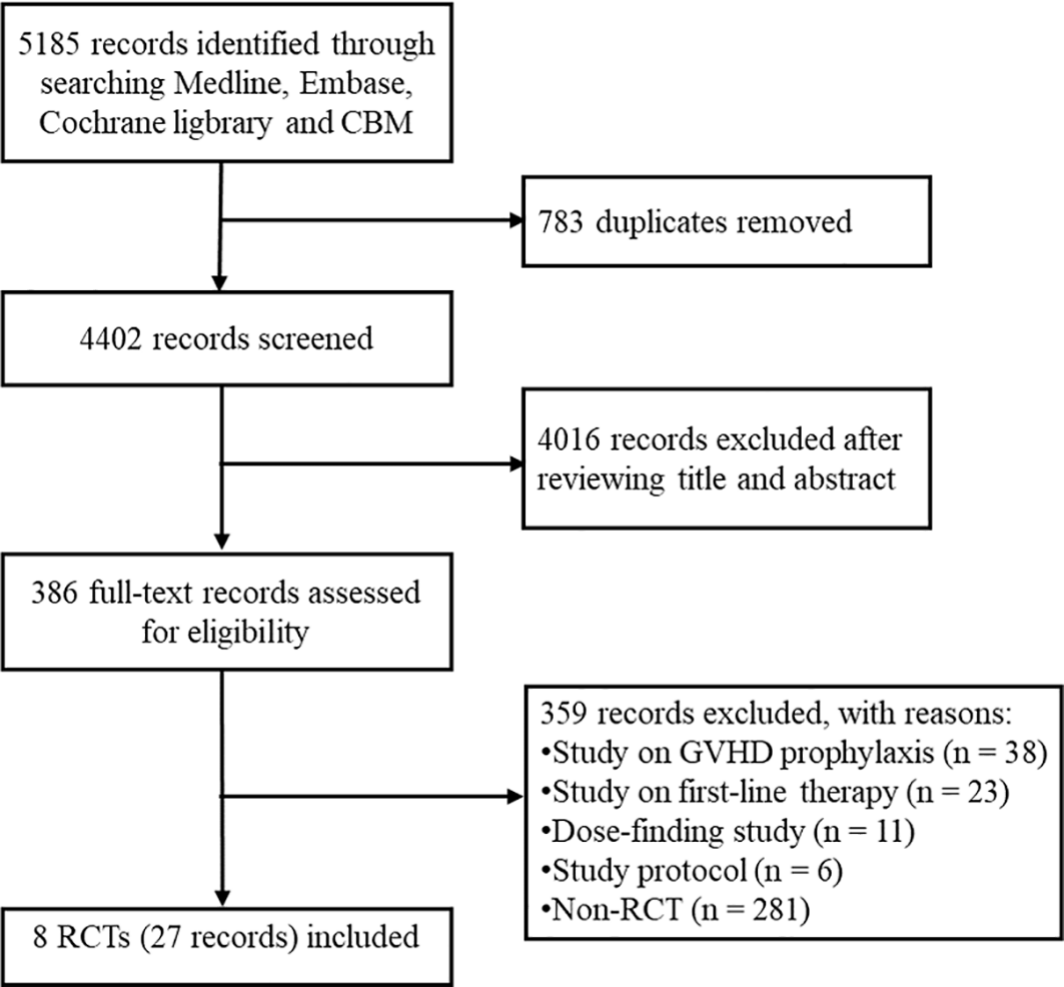
The flow chart of study selection. CBM, China Biology Medicine; GVHD, graft-versus-host disease; RCT, randomized controlled trial.

The databases search retrieved 5185 potentially relevant records. We removed 783 duplicates and excluded 4016 irrelevant records based on title and abstract, the remaining 386 records were included for further screening. Subsequently, we excluded 38 studies on GVHD prophylaxis, 23 studies on first-line therapy of aGVHD, 11 dose-finding studies, 6 protocol and 281 non-RCT studies. Ultimately, 27 records (19 conferences abstract and 8 full-text article) for 8 eligible RCTs were included for the systematic review (21–29). The flow chart of study selection is provided in **Figure 1**.

The characteristics of the 8 included RCTs are summarized in **Table 1**. The specific definition of steroids-refractory aGVHD in each included trail is summarized in **Supplementary Table 1**. A broad range of second-line therapy strategies for steroids-refractory aGVHD are investigated, the dosage of all regimens are provided in **Supplementary Table 2**. Three trials investigated the effects of adding MSCs to other second-line therapy regimens (including MMF, ATG, infliximab, etanercept, daclizumab, pentostatin, denileukin diftitox, ECP, alemtuzumab, and basiliximab plus cyclosporine or tacrolimus) (21–23). One trial (the REACH2 trial) compared the efficacy and safety of ruxolitinib with other best available care (investigator’s choice from ATG, ECP, MSCs, LD-MTX, MMF, everolimus, sirolimus, etanercept, or infliximab) (24). Other trials compared high-dose methylprednisolone (HD-MP) plus OKT3 (a murine anti-CD3 monoclonal antibody) or ATG with HD-MP alone, or compared ATG with inolimomab (a monoclonal antibody against IL-2R) or ABX-CBL (a murine monoclonal antibody against CD147) (25–29). The primary endpoints were largely different, including ORR (at day 28, 30 or 100), durable complete response, therapy success rate, and OS at day 180. The Median follow-up ranges from 180 days to 58.4 months (**Table 1**).

**Table 1:** Characteristics of included trials and patients.

Study	Study design	Treatment group	No. of patients	Age^*^	Gender (Male%)	Underlying disease	Donor	Graft source	aGVHD grade at baseline	Median follow-up
Zhao 2022 (21)	Phase 3 RCT, open-label, multicenter (NCT02241018)	MSC + Second-line therapy	99	28 (16–59)	63%	AML: 39.4%; ALL: 45.5%; Others: 15.2%	HLA matched: 51.5%; HLA mismatched: 48.5%	PBSC: 53.5%; PBSC + BM: 46.5%	II: 36.4%; III: 41.4%; IV: 22.2%	19.8 months
		Second-line therapy	99	29 (16–57)	57%	AML: 49.5%; ALL: 43.4%; Others: 7.1%	HLA matched: 51.5%; HLA mismatched: 48.5%	PBSC: 57.6%; PBSC + BM: 42.4%	II: 37.4%; III: 44.4%; IV: 18.2%	12.3 months
Oosten 2022 (22)	Phase 3 RCT, double-blind, multicenter (HOVON-113 trial)	MSC + Second-line therapy	20	NA	NA	NA	NA	NA	II: 12%; III: 68%; IV: 20%	24.0 months
		Second-line therapy	21	NA	NA	NA	NA	NA		
Kebriaei 2020 (23)	Phase 3 RCT, double-blind, multicenter (NCT00366145)	MSC + Second-line therapy	163	43.8 ± 16.6	56%	NA	Unrelated: 57%; Related: 43%	BM: 12%; PBSC: 78%; CB: 10%	B: 23%; C: 50%; D: 27%	180 days
		Second-line therapy	80	40.0 ± 18.1	59%	NA	Unrelated: 58%; Related: 42%	BM: 17%; PBSC: 70%; CB: 12%	B: 26%; C: 57%; D: 17%	180 days
Zeiser 2020 (24)	Phase 3 RCT, open-label, multicenter (REACH2 trail, NCT02913261)	Ruxolitinib	154	53 (12–73)	60%	AML: 37.7%; ALL: 16.2%; CML: 3.9%; MDS: 16.9%; NHL: 5.8%; Others: 19.2%	NA	BM: 12.3%; PBSC: 87.0%; CB: 0.6%	I: 1.3%; II: 32.5%; III: 44.2%; IV: 19.5%	5.04 months
		Investigator’s choice	155	54 (13–71)	59%	AML: 40.6%; ALL: 10.3%; CML: 1.3%; MDS: 18.7%; NHL: 12.3%; Other: 16.8%	NA	BM: 19.4%; PBSC: 76.1%; CB: 4.5%	I: 0%; II: 34.8%; III: 43.9%; IV: 20.6%	3.58 months
Socie 2017, 2019 (25, 26)	Phase 3 RCT, open-label, multicenter (EudraCT 2007-005009-24)	Inolimomab	49	46.2 ± 12.6	45%	AML: 29%; ALL: 14%; MDS: 8%; CLL: 8%; Lymphoma: 16%; Other: 25%	MSD: 31%; MUD: 63%; MMUD: 6%	PBSC: 81.6%; BM: 18.4%	I: 2%; II: 22.4%; III: 63.3%; IV: 12.2%	58.4 months
		ATG	51	47.1 ± 12.96	51%	AML: 20%; ALL: 12%; MDS:14%; CLL: 12%; Lymphoma: 20%; Other: 22%	MSD: 39%; MUD: 59%; MMUD: 2%	PBSC: 76.5%; BM: 23.5%	I: 6.1%; II: 12.2%; III: 65.3%; IV: 16.3%	58.4 months
Knop 2007 (27)	RCT, multicenter (by EBMT)	HD-MP + OKT3	40	40 (19–65)	70%	AML/MDS: 45%; ALL: 2.5%; CML: 30%; Other: 22.5%	MSD: 30%; MUD: 52.5%; MMUD: 17.5%	PBSC: 70%; BM: 30%	II: 25%; III: 62.5%; IV: 12.5%	NA
		HD-MP	40	39 (19–56)	65%	AML/MDS: 40%; ALL: 12.5%; CML: 32.5%; Other: 10%	MSD: 50%; MUD: 35%; MMUD: 15%	PBSC: 62.5%; BM: 37.5%	II: 25%; III: 62.5%; IV: 12.5%	NA
MacMillan 2007 (28)	Phase 2/3 RCT, multicenter, open-label	ABX-CBL	48	38 (2-65)	65%	AML: 29%; ALL: 6%; CML: 15%; MDS: 12%; Lymphoma: 21%; Other: 17%	MRD: 40%; MMRD: 2%; MUD: 44%; MMUD: 14%	NA	B/C: 85%; D: 15%	180 days
		ATG	47	39 (2-65)	57%	AML: 19%; ALL: 17%; CML: 26%; MDS: 8%; Lymphoma: 17%; Other: 13%	MRD: 47%; MMRD: 4%; MUD: 34%; MMUD: 15%	NA	B/C: 91%; D: 15%	180 days
Van Lint 2006 (29)	RCT, multicenter (by GITMO)	HD-MP + ATG	27	36 (2–63)	56%	AML/ALL: 44%; CML: 26%; Other: 30%	HLA-identical sibling: 52%; Unrelated: 48%	NA	I: 15%; II: 59%; III: 26%	1134 days
		HD-MP	34	33 (2–64)	50%	AML/ALL: 53%; CML: 35%; Other: 12%	HLA-identical sibling: 47%; Unrelated: 53%	NA	I: 21%; II: 56%; III: 24%	1134 days

^*^Age is presented as mean ± standard deviation or median with range.

ABX-CBL, a murine monoclonal antibody against CD147; aGVHD, acute graft-versus-host disease; ALL, acute lymphoblastic leukemia; AML, acute myeloid leukemia; ATG, anti-thymocyte globulin; BM, bone marrow; CLL, chronic lymphoblastic leukemia; CML, chronic myeloid leukemia; CR, complete response; DCR, durable complete response; EBMT, European Group for Blood and Marrow Transplantation; ECP, extracorporeal photopheresis; GITMO, Gruppo Italiano Trapianto Midollo Osseo; HD-MP, high-dose methylprednisolone; HL, Hodgkin lymphoma; HLA, human leukocyte antigen; MDS, myelodysplastic syndrome; MMF, mycophenolate mofetil; MMUD, mismatched unrelated donor; MP, methylprednisolone; MRD, matched related donor; MSC, mesenchymal stem cell; MSD, matched sibling donor; MRD, matched unrelated donor; NA, not available; NHL, non-Hodgkin lymphoma; OKT3, a murine monoclonal antibody against CD3; ORR, overall response rate; PBSC, peripheral blood stem cells; RCT, randomized controlled trial; SR, steroid-refractory.

A total of 1127 patients were included and most of them were diagnosed with hematological malignancies (**Table 1**). Patient’s age, gender and grade of aGVHD were well matched between the experimental group and control group in 7 of the included trials that with enough information. As for donor type, 5 trials with relevant information included both related and unrelated donors. Graft sources were reported in 5 trials, which included both bone marrow (BM) and peripheral blood stem cells (PBSCs). Two trials included small proportions of patients (0.6%-12%) receiving cord blood (CB) transplantation (23, 24).

The results of risk of bias assessment suggest that random sequence generation is adequate in 3 trials, allocation sequence concealment is adequate in 2 trials, whereas the other trials did not provide sufficient information to evaluate selection bias (**Supplementary Figure 1**). As for blinding of participants and personnel, 2 trials are double-blind, 4 trials are open-label. Outcome assessments were performed in blinded manner in 3 trials. All of the included trials are free from attrition bias, reporting bias and any other bias except for one trial that only reported as a conference abstract and did not provide sufficient information to evaluate.

### Overall response rate

**Figure 2:**
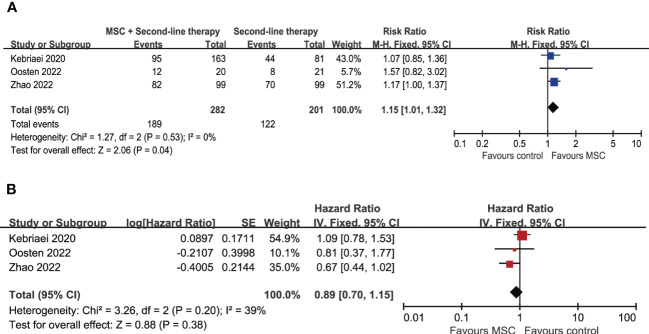
Forest plot for meta-analysis including MSC-related trials. **(A)** Overall response rate at day 28. **(B)** Overall survival. MSC, mesenchymal stem cells.

**Figure 3:**
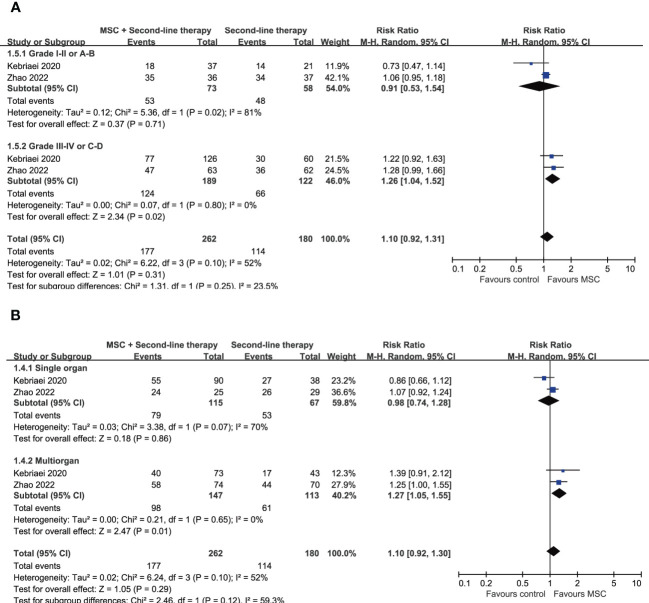
Forest plot for subgroup analysis on ORR at day 28 according to **(A)** the grade of aGVHD and **(B)** the number of involved organs. aGVHD, acute graft-versus-host disease; MSC, mesenchymal stem cells.

Treatment outcomes including overall response rate (ORR), complete respou rate and overall survival of all included trials are summarized in **Table 2**. The ORR at day 28 were reported in 4 trials, of which 3 trials investigated the effects of adding MSCs to other second-line therapy regimens (21–23). The ORR at day 28 of those second-line therapy regimens without MSCs ranges from 36.4% to 70.7%, addition of MSCs increased it to 47.6-82.8% (**Table 2**). The result of meta-analysis suggested that adding MSCs to other second-line therapy regimens is associated with significant increase of ORR at day 28 (RR = 1.15, 95% CI = 1.01–1.32, *P* = 0.04; **Figure 2A**). Subgroup analyses were performed based on patients’ age, grade of aGVHD, involved organ, and the number of involved organs, suggests that the addition of MSCs is associated with significant improve of ORR at day 28 in patients with severe (grade III–IV or grade C–D) aGVHD (RR = 1.26, 95% CI = 1.04–1.52, *P* = 0.02) and patients with multiorgan involved (RR = 1.27, 95% CI = 1.05–1.55, *P* = 0.01) although the subgroup differences were not statistically significant (**Figures 3A, B**). Subgroup analyses according to patients’ age and involved organ did not show any statistically significant results (**Supplementary Figures 2**, **3**).

**Table 2:** Treatment outcomes of included trials.

Study	Treatment group	ORR	CR rate	OS	Relapse of underlying disease	Common AEs
Zhao 2022 (21)	MSC + Second-line therapy	82.8% (82/99) (at day 28)^†^	56.6% (56/99) (at day 28)^†^	6-month OS: 68.7%; 1-year OS: 67.1%; 3-year OS: 63.4%	3-year CIR: 10.1%	Hematologic (grade ≥ 3): 37.4%^†^; Infection (grade ≥ 3): 65.7%^†^
	Second-line therapy (Basiliximab + calcineurin inhibitor)	70.7% (70/99) (at day 28)	40.4% (40/99) (at day 28)	6-month OS: 60.6%; 1-year OS: 54.8%;	3-year CIR: 13.5%	Hematologic (grade ≥ 3): 53.5%; Infection (grade ≥ 3): 78.8%
Oosten 2022 (22)	MSC + Second-line therapy	60% (12/20) (at day 28)	NA	1-year OS: 45%	NA	Infection grade 3: 22%; Infection grade 4: 39%
	Second-line therapy (MMF)	38% (8/21) (at day 28)	NA	1-year OS: 33%	NA	Infection grade 3: 25%; Infection grade 4: 45%
Kebriaei 2020 (23)	MSC + Second-line therapy	58.3% (95/163) (at day 28)	36.8% (60/163) (DCR^*^)	180-day OS: 34%	180-day CIR: 8%	All infection: 88.3%; Edema peripheral: 35.6%; Abdominal pain: 22.7%; Thrombocytopenia: 22.1%
	MSC + ATG	61.8% (21/34)	41.2% (14/34)			
	MSC + MMF	63.0% (17/27)	48.2% (13/27)			
	MSC + Infliximab	62.1% (18/29)	44.8% (13/29)			
	MSC + Etanercept	47.6% (10/21)	33.3% (7/21)			
	MSC + Daclizumab	73.3% (11/15)	33.3% (5/15)			
	Second-line therapy	54.3% (44/81) (at day 28)	32.1% (26/81) (DCR^*^)	180-day OS: 42%	180-day CIR: 9.9%	All infection: 81.5%; Edema peripheral: 33.3%; Abdominal pain: 17.3%; Thrombocytopenia: 22.2%
	ATG	58.8% (10/17)	23.5% (4/17)			
	MMF	50.0% (8/16)	31.3% (5/16)			
	Infliximab	53.9% (7/13)	23.1% (3/13)			
	Etanercept	36.4% (4/11)	45.5% (5/11)			
	Daclizumab	54.6% (6/11)	54.6% (6/11)			
Zeiser 2020 (24)	Ruxolitinib	62% (96/154) (at day 28)^†^	34% (53/154) (at day 28)^†^	6-month OS: 59.5%; 1-year OS: 48.7%	18-month CIR: 13%	Thrombocytopenia: 33%^†^; Anemia: 30%; CMV infection: 26%; Infection (grade ≥ 3): 22% (up to day 28)
	Investigator’s choice	39% (61/155) (at day 28)	19% (30/155) (at day 28)	6-month OS: 50.4%; 1-year OS: 43.6%	18-month CIR: 19%	Thrombocytopenia: 18%; Anemia: 28%; CMV infection: 21%; Infection (grade ≥ 3): 19% (up to day 28)
	ATG	30% (6/20)	15% (3/20)			
	Etanercept	45.5% (10/22)	27.3% (6/22)			
	ECP	43.9% (18/41)	19.5% (8/41)			
	Infliximab	35.3% (6/17)	11.8% (2/17)			
	MSC	60% (9/15)	20% (3/15)			
	MMF	32% (8/25)	16% (4/25)			
Socie 2017, 2019 (25, 26)	Inolimomab	28.5% (Therapy success rate^#^)	NA	1-year OS: 47%; OS after LTFU: 30.6%^†^	1-year CIR: 13%	Viral, bacterial, fungal infection: 78%, 82%, 35%
	ATG	21.5% (Therapy success rate^#^)	NA	1-year OS: 40%; OS after LTFU: 19.6%	1-year CIR: 6%	Viral, bacterial, fungal infection: 92%, 84%, 37%
Knop 2007 (27)	HD-MP + OKT3	53% (21/40) (at day 100)	NA	1-year OS: 45%;	NA	CRS: 60%; Hyperglycemia: 43%; Viral, bacterial, fungal infection: 35% ^†^, 10%, 10%
	HD-MP	33% (13/39) (at day 100)	NA	1-year OS: 36%;	NA	Hyperglycemia: 49%; Viral, bacterial, fungal infection: 72%, 27%, 10%
MacMillan 2007 (28)	ABX-CBL	56% (27/48) (at a median of 22 days)	29% (14/48) (at a median of 77 days)	180-day OS: 35.4%	NA	Infection and infestations: 97.8%; Blood culture positive: 56.5%; Hypertension: 30.4%; Pneumonia: 33%^†^
	ATG	57% (27/47) (at a median of 28 days)	32% (15/47) (at a median of 78 days)	180-day OS: 44.7%	NA	Infection and infestations: 100%; Blood culture positive: 45.7%; Hypertension: 28.3%; Pneumonia: 65%
Van Lint 2006 (29)	HD-MP + ATG	55% (15/27) (at day 30)	33% (9/27) (at day 30)	OS at the end of follow-up: 34%	NA	NA
	HD-MP	48% (16/34) (at day 30)	24% (8/34) (at day 30)	OS at the end of follow-up: 36%	NA	NA

^*^DCR, durable complete response, defined as CR for at least 28 consecutive days within the first 100 days after enrollment.

^#^Therapy success was defined as overall survival at 1 year without replacement of the baseline allocated treatment.

^†^Results with significant difference in comparison with control group (P < 0.05).

ABX-CBL, a murine monoclonal antibody against CD147; ATG, anti–thymocyte globulin; CIR, cumulative incidence of relapse; CR, complete response; CRS, cytokine release syndrome; DCR, durable complete response; ECP, extracorporeal photopheresis; HD-MP, high-dose methylprednisolone; LTFU, long-term follow up (with a median follow-up of 58.4 months); MMF, mycophenolate mofetil; MP, methylprednisolone; MSCs, mesenchymal stem cells; NA, not available; OKT3, a murine monoclonal antibody against CD3; ORR, overall response rate; OS, overall survival; SAEs, serious adverse events.

Another trial (the REACH2 trial) reporting ORR at day 28 as primary endpoint compared the efficacy of ruxolitinib with investigator’s choice from other second-line therapy regimens, including ATG, etanercept, ECP, infiliximab, MSCs and MMF (24). The ORR at day 28 of these regimens ranges from 30% to 60%, leading to a total ORR of 39% (**Table 2**). Ruxolitinib is associated with a significantly higher ORR at day 28 (62% versus 39%, *P* < 0.001) and durable overall response at day 56 (40% versus 22%, *P* < 0.001) in comprison with investigator’s choice.

The other 4 trials reported ORR at different time phase or 1-year therapy success rate (**Table 2**). Two trials investigated the efficacy of HD-MP plus ATG or anti-CD3 antibody OKT3, suggested that HD-MP plus ATG did not significantly improve ORR at day 30 over HD-MP alone (55% versus 48%; *P* = 0.3), and HD-MP plus OKT3 did not significantly improve ORR at day 100 over HD-MP alone (53% versus 33%; *P* = 0.06) (27, 29). One trial compared the efficacy of anti-CD147 antibody ABX-CBL with ATG, reported an ORR of 56% for the ABX-CBL group at a median of 22 days (range 7–72 days) and 57% for the ATG group at a median of 28 days (range 2–50 days), no significant difference was observed (28). The other trial compared inolimomab with ATG and reported 1-year therapy success rate as the primary endpoin (defined as the overall survival at 1 year without replacement of the baseline allocated treatment), and no significant differences was observed (28.5% versus 21.5%; *P* = 0.188) (25, 26).

### Complete response rate

The complete response (CR) rate were reported in 5 of the included trials (reviewed in **Table 2**). One of the 2 trials investigating the effects of adding MSCs with data regarding CR rate showed that the addition of MSCs led to a significantly improvement in CR rate at day 28 (56.6% versus 40.4%, *P* = 0.02), whereas another trial reported durable complete response (DCR, defined as CR for at least 28 consecutive days within the first 100 days after enrollment) as primary endpoint and no significant difference was observed (35% versus 30%, *P* = 0.42) (21, 23). The REACH2 trial reported that ruxolitinib led to significantly higher CR rate at day 28 in comparison with investigator’s choice from other second-line therapy (34% versus 19%, *P* = 0.004) (24). As for the trial comparing HD-MP plus ATG with HD-MP alone, CR rate at dat 30 was reported, and no significant difference was observed (33% versus 24%, *P* = 0.40) (29). For ABX-CBL versus ATG, The CR rates are 29% at a median of 77 days (range 14-77 days) in ABX-CBL group versus 32% at a meidan of 78 days (range 21-88 days) in ATG group (28).

### Overall survival and relapse of underlying disease

After a median follow-up ranging from 180 days to 58.4 months, the 8 included trials reported overall survival (OS) at 180 days, 6 months, 1 year, 18-months, 3 years or the end of follow-up (summarized in **Table 2**). Meta-analysis of the 3 trials investigating the effects of adding MSCs to other second-line therapy regimens suggests that there is no significant difference in OS (HR = 0.89, 95% CI = 0.70–1.15, *P* = 0.38; **Figure 2B**). As for other comparisons, ruxolitinib did not lead to significant improvement in median OS (11.1 months versus 6.5 month; HR = 0.83, 95% CI = 0.60–1.15) but significantly prolonged median failure-free survival (5 months versus 1 month; HR = 0.46, 95% CI = 0.35–0.60); inolimomab showed more favorable OS over ATG (HR = 0.57, 95% CI = 0.35–0.95, *P* = 0.03) after long-term follow-up (up to 104 months, with a median 58.4 months) (25). The other trials did not report significant differences in OS between experimental group and control group (**Table 2**). The cumulative incidence of relapse of underlying disease (at 180 days, 1 year, 18-months, or 3 years) are provided in 4 trials, no significant difference is reported (**Table 2**).

### Safety

The most common adverse events (AEs) reported in the 8 included trials are hematologic toxicities and infection (summarized in **Table 2**). One trial investigating the effects of adding MSCs to other second-line therapy regimens suggested that the MSCs group is associated with lower incidences of grade 3–4 hematologic toxicities (37.4% versus 53.5%, *P* = 0.022) and grade 3–4 infection (65.7% versus 78.8%, *P* = 0.039) (21). The REACH2 trial reported that ruxolitinib is associated with higher rates of thrombocytopenia (33% versus 18%, *P* = 0.003) compared with the investigator’s choice group (24). In comparison with ATG, inolimomab showed slightly lower incidences of viral infection (78% versus 92%, *P* = 0.05), and ABX-CBL showed lower incidences of pneumonia (33% versus 65%, *P* = 0.002). In comparison with HD-MP alone, HD-MP plus OKT3 is associated with lower risk of viral infection (35% versus 72%, *P* = 0.001), but caused cytokine release syndrome (CRS) at a rate around 60%. Other reported data in about AEs did not show any significant difference between experimental group and control group.

Meta-analyses results show that the MSCs plus other second-line therapy regimens is associated with similar risk of serious AEs (RR = 0.99, 95% CI = 0.88–1.12, *P* = 0.91), serious AEs with outcome of death (RR = 0.95, 95% CI = 0.56–1.59, *P* = 0.83), and infection-related death (RR = 1.17, 95% CI = 0.51–2.66, *P* = 0.71) in comparison with the control group (**Supplementary Figure 4**).

## Discussion

Steroid-refractory aGVHD is a life-threatening conditioning resulting in mortality rates higher than 90%. Treatment of steroid-refractory aGVHD remains a great challenge in clinical practice, the standard strategy still has not been established. This study performed a systematic review and meta-analysis of RCTs comparing the efficacy and safety of different second-line therapy regimens in patients with steroid-refractory aGVHD undergoing allo-HSCT. The reported treatment outcomes (including overall response rate, complete response rate, overall survival and main safety outcomes) of all second-line therapy in 8 eligible RCTs were comprehensively reviewed, and meta-analysis of 3 trials about MSCs was performed. We find that the addition of MSCs to other second-line therapy regimens are associated with significantly improved ORR at day 28, especially in patients with severe (grade III–IV or grade C–D) aGVHD and patients with multiorgan involved. The overall survival, risk of serious AE and serious AE-related death are similar between the MSCs and non-MSCs group. As for other comparisons, ruxolitinib showed significantly higher ORR and CR rate than other best available treatments; inolimomab was associated with superior long-term OS versus ATG. These evidences suggest that MSCs-contained second-line therapy regimens and ruxolitinib are asscociated with favorable efficacy outcomes in patients with steroid-refractory aGVHD.

The benefits from MSCs in the prophylaxis and treatment of immune-related disorders are widely investigated due to its multipotency and immunomodulatory properties (30). Various mechanisms are involved in the immunoregulating activities of MSCs, such as suppressing the proliferation and activation of CD4^+^ T cells, increasng the percentage of regulatory T cells, and modifying the cytokine secretion profile of different immune cells (30–32). In patients undergoing allo-HSCT, co-transplantation of MSCs can promotes engraftment and reduce the risk of severe aGVHD (33–35). For the treatment of GVHD, MSCs are often administrated in combination with other regimens, and the efficacy varies greatly due to the differences in MSCs source and dosage, disease characteristics and the combined regimens (35–38). Commercial MSCs product Remestemcel-L (Prochymal) was approved pediatric patients in Canada and New Zealand, and Temcell was approved in Japan, but the role of MSCs in the treatment of steroid-refractory aGVHD still require to be established (38). A meta-analysis of 13 non-randomised studies obtained an ORR of 72% and a 6-month survival of 63% for MSCs treatment in patients with steroid-refractory aGVHD (37). Afterwards, three RCTs were published and our meta-analyses results suggested that the addition of MSCs to other second-line therapy is associated with significant improvement in ORR, especially in patients with severe disease and multiorgan involvement (21–23). An ongoing phase 3 RCT (NCT04629833) comparing MSCs with best available therapy (BAT) in patients with steroid-refractory aGVHD will be helpful to further establish the benefits of MSCs (39). However, the source and dosage of MSCs, and the choice of combined regimens vary across these 3 published RCTs. Considering the combined regimens, the highest ORR (82.8%) and CR rate (56.6%) at day 28 were reported when MSCs are administrated in combination with basiliximab and calcineurin inhibitor (reviewed in **Table 2**), further studies are required to determine the optimal treatment strategy of MSCs in patients with different characteristics.

Ruxolitinib, and selective inhibitor of Janus kinase (JAK) 1 and JAK2, is the only approved drugs for the treatment of steroid-refractory aGVHD by US Food and Drug Administration (FDA). The JAK1/2 signaling plays an important role in the mechasims of GVHD through mediating the activation and proinflammatoy cytokines release of T cell, neutrophils and dendritic cells (DCs) (40, 41). Preclinical studies suggested that ruxolitinib can effectively inhibit JAK1/2 signaling, and ameliorate both acute and chronic GVHD while preserving graft-versus-tumor activity (42, 43). For patients with steroid-refractory aGVHD, ruxolitinib orally given at a dose of 5–10 mg achieved a ORR at day 28 of 55–82% with acceptable toxicity (24, 44, 45). The multicenter RCT (REACH2 trial) reported that compared with a control group incorporting a series of seond-line therapy regimens, ruxolitinib led to significantly higher ORR at day 28, higher durable overall response at day 56 and superior failure-free survival (24). A recently published meta-analysis including both prospective and retrospective studies reported an ORR of 77% and a 6-month survival of 63.9% for ruxolitinib in patients with steroid-refractory aGVHD, seem to be comparable to that of MSCs- or basiliximab-based second-line therapy regimens (46). RCT directly comparing the efficacy and safety of ruxolitinib with specific second-line regimens such as MSCs- and basiliximab-based regimen is unavailable, further trials and integrated studies are required to validate the advantages of ruxolitinib over other srategies.

Antagonists of IL-2R represent another attractive option for the therapy of steroid-refractory aGVHD. Blocking IL-2 signaling with IL-2R antagonists such as antibodies against IL-2R alpha chain (CD25) (basiliximab, daclizumab and inolimomab) and denileukin difititox (a recombinant fusion protein composed of IL-2 fragment and diphtheria toxin) show promising activity in steroid-refractory aGVHD (47–50). The effects of combining IL-2R antagonists with inhibitors of TNF-α signaling (etanercept and infliximab) were also investigated (51–54). According to the 2 RCTs included in our review, basiliximab achieved an ORR at day 28 of 70.7% and 82.8% with or without MSCs respectively, inolimomab achieved a 1-year therapy success rate of 28.5% and an advantage in long-term survival over ATG (21, 25). No RCT comparing the efficacy of these four IL-2R antagonists is available, a meta-analysis pooling data from both prospective and retrospective studies suggested that basiliximab-based therapy is associated the highest ORR of 81%, followed by 71% for daclizumab, 56% for denileukin difititox and 54% for inolimomab (55). Well-designed prospective studies will be helpful to validate the advantages of basiliximab over other IL-2R antagonists or other regimens in the therapy of steroid-refractory aGVHD.

There are several regimens of other mechanisms that were not involved in included trials, such as the anti-α4β7 integrins monoclonal antibody vedolizumab and the anti-interleukin-6 receptor (IL-6R) monoclonal antibody tocilizumab. A systematic review and meta-analysis have evaluated the efficacy of vedolizumab in the treatment of gastrointestinal aGVHD and obtained a pooled long-term ORR higher than 70% (56). As for steroid-refractory gastrointestinal aGVHD, several retrospective studies of small sample size were published and the reported ORR ranges from 45% to 79% (57–59). The efficacy of tocilizumab in steroid-refractory aGVHD was also only evaluated in a few retrospective studies with small sample size, reporting CR rate of 40%-60% (60, 61). Further studies are warranted to determine the efficacy and safety of these regimens with different mechanisms.

There are several limitations in this study. First of all, the number of published RCTs regarding therapy of steroid-refractory aGVHD is limited. Secondly, the comparative statistical analysis failed to include all investigated regimens since the regimens applied in the control groups varied largely across included tirals, including HD-MP, ATG and an incoporation of a series of best available choice, making it impractical to integrated evidences with traditional or network-meta-analysis. In future research, unification of control regimen will be helpful to increase the application of analyzed results for guiding clinical practice. Thirdly, the reported efficacy outcomes also varied across included tirals, making it impractical to integrate data from different trials. We choose ORR at day 28 as the primary outcome since it is proposed as the best endpoint for aGVHD therapeutic trials in predicting transplantation-related mortality (8). However, the ORR at day 28 are only reported in 4 included RCTs. Lastly, long-term follow-up data, which we think are very important for patients undergoing allo-HSCT, are absent in several included studies.

In conclusion, this systematic review and meta-analysis of RCTs suggests that MSCs-contained second-line therapy regimens and ruxolitinib are associated with favorable efficacy outcomes in patients with steroids-refractory aGVHD, but further well-designed RCTs and integrated studies are still required to determine the optimal treatment.

## Data availability statement

The original contributions presented in the study are included in the article/**Supplementary Material**. Further inquiries can be directed to the corresponding authors.

## Author contributions

SX, YZ, XL and JC contributed to the study conception and design, supervised the study and made critical revision in the manuscript. CL, XH and LW performed database searching, study selection, data extraction, statistical analyses and manuscript writing. GW, YH, YD, and ZH helped with data extraction, data checking and data interpretation. All authors contributed to the article and approved the submitted version.
