# Supplementary figures and images for: Second-line therapy for patients with steroid-refractory aGVHD: systematic review and meta-analysis of randomized controlled trials
Source: Front Immunol. 2023 Jun 20;14:1211171. doi: 10.3389/fimmu.2023.1211171 (PMC10318925; doi:10.3389/fimmu.2023.1211171)

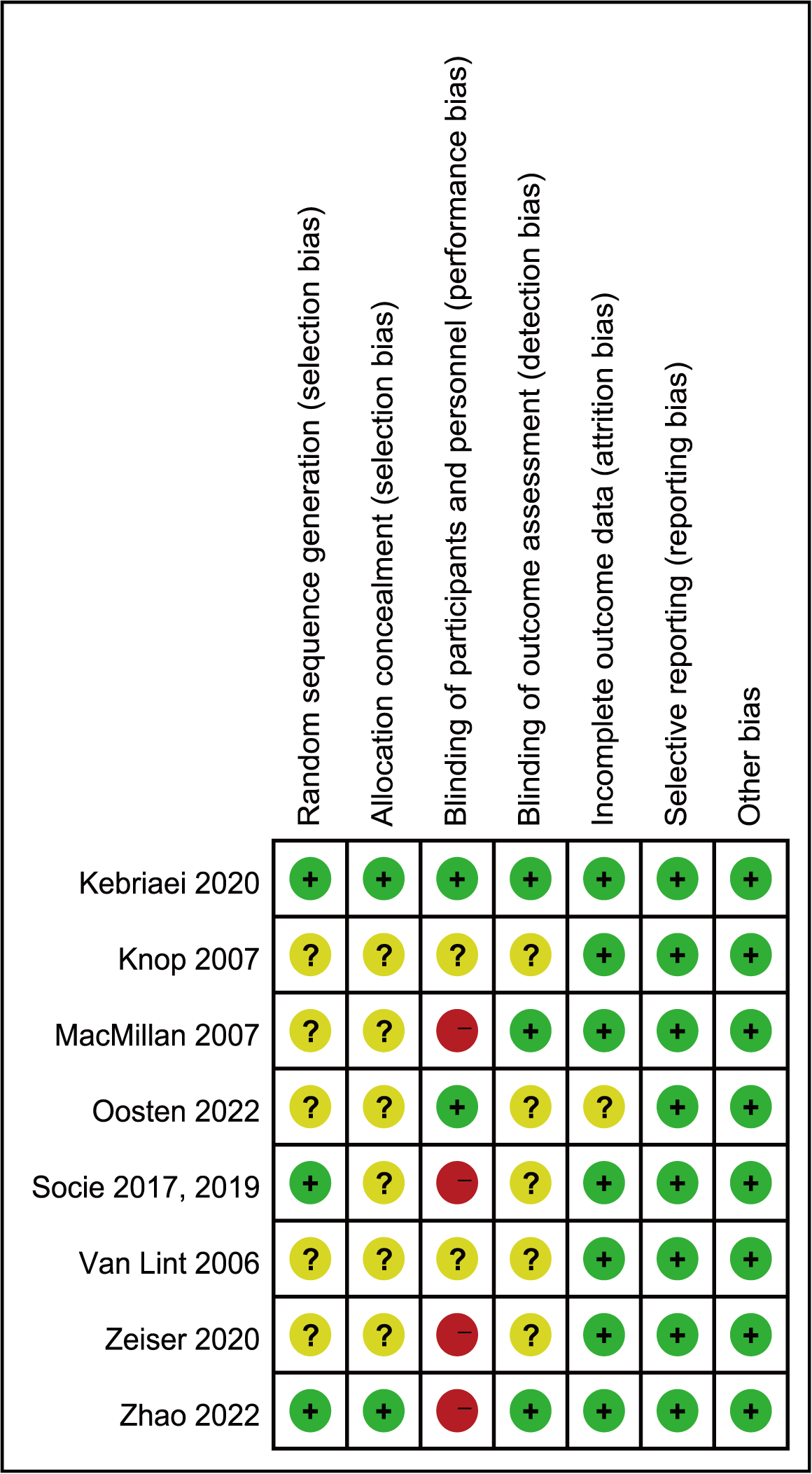

Supplement: Supplementary file 1 [file Image_1.tif]

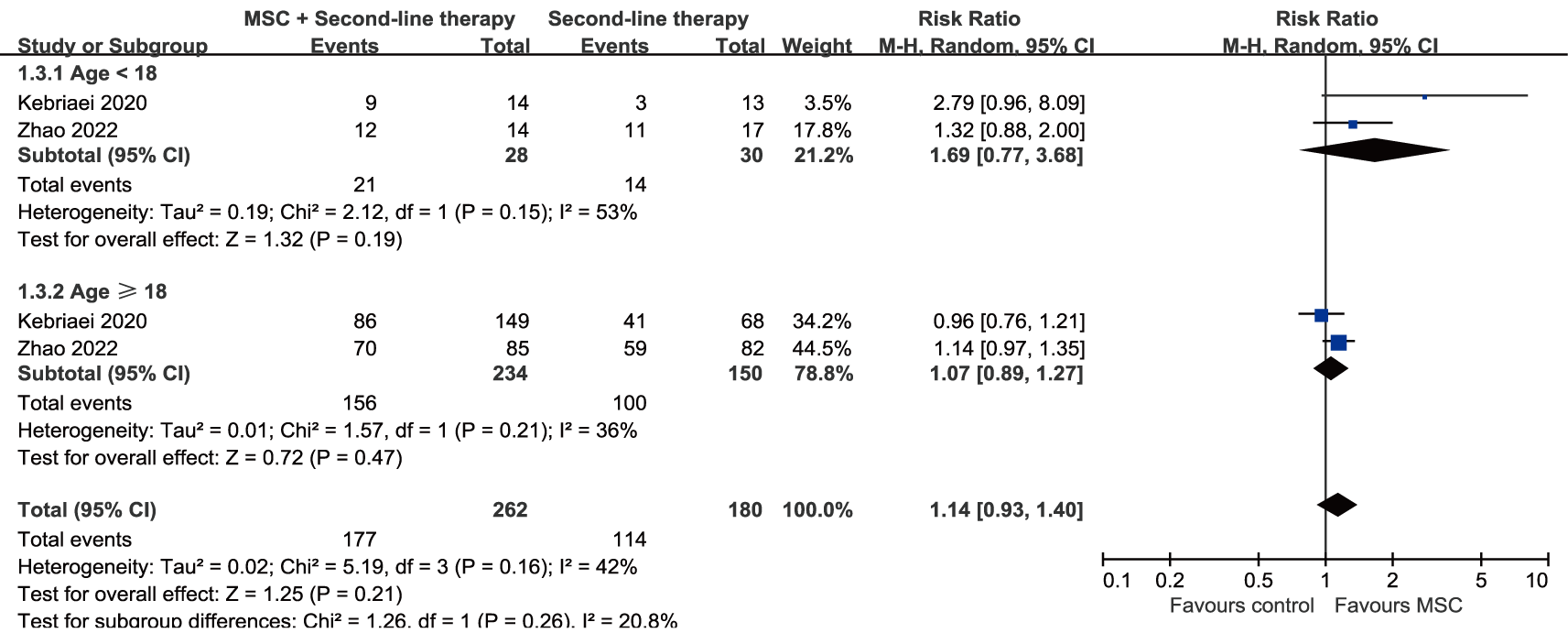

Supplement: Supplementary file 2 [file Image_2.tif]

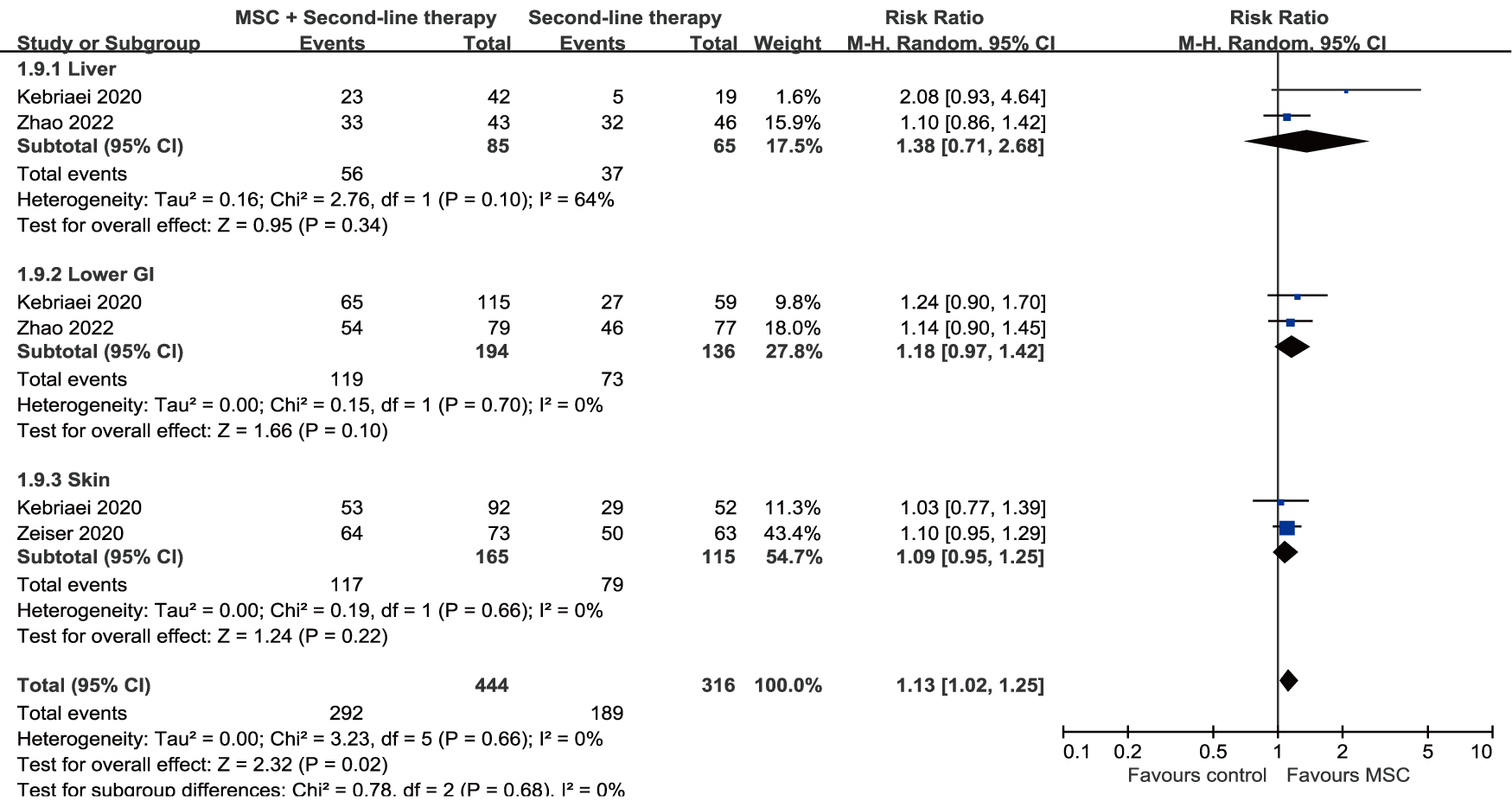

Supplement: Supplementary file 3 [file Image_3.tif]

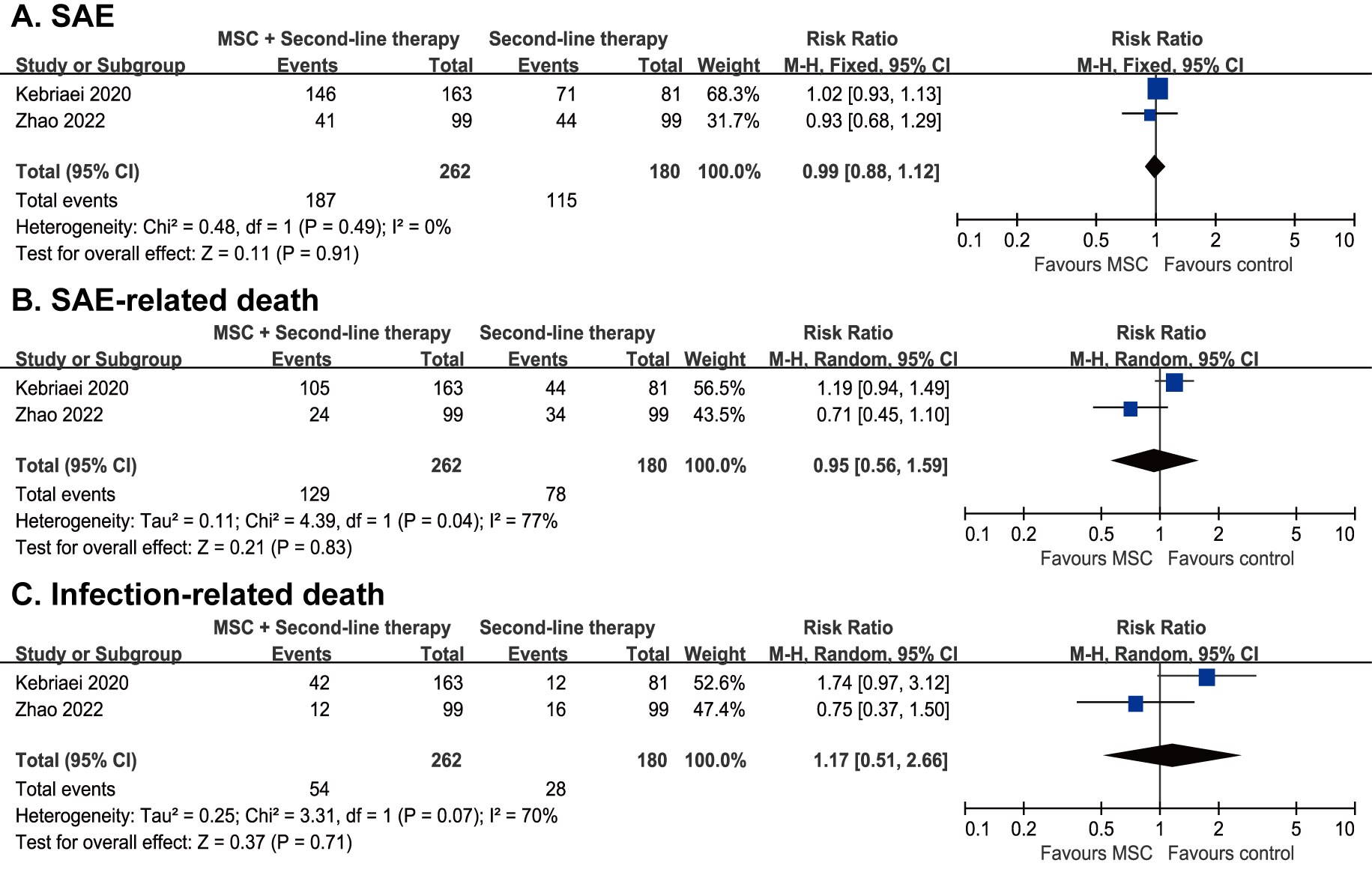

Supplement: Supplementary file 4 [file Image_4.tif]
