# Supplementary material for: Second-line therapy for patients with steroid-refractory aGVHD: systematic review and meta-analysis of randomized controlled trials
Source: Front Immunol. 2023 Jun 20;14:1211171. doi: 10.3389/fimmu.2023.1211171 (PMC10318925; doi:10.3389/fimmu.2023.1211171)
Supplement: Supplementary file 5 [file Table_1.docx]

**Supplementary Table 1. The specific definition of steroids-refractory aGVHD in included studies.**

| **Study** | **Definition of steroids-refractory aGVHD** |
| --- | --- |
| Zhao 2022 | aGVHD worsening after 3 days of therapy onset with ≥ 2 mg/kg/day of methylprednisolone or equivalent, or failure to improve after 7 days of treatment initiation; or treatment failure during steroid taper (i.e., an increase in the methylprednisolone dose to ≥ 2 mg/kg/day or equivalent or an inability to taper the dose to < 0.5 mg/kg/day of methylprednisolone or equivalent for a minimum of 7 days). |
| Kebriaei 2020 | Failure to improve by at least 1 grade after at least 3 days and no greater than 2 weeks of at least 1 mg/kg/d methylprednisolone or equivalent. |
| Zeiser 2020 | Disease progression on the basis of organ assessment after at least 3 days of high-dose systemic glucocorticoid therapy, with or without calcineurin inhibitors; a lack of response (absence of partial response or better) after 7 days; or treatment failure during glucocorticoid taper (i.e., an increase in the methylprednisolone dose to ≥ 2 mg per kilogram of body weight per day [or equivalent ≥ 2.5 mg per kilogram per day of prednisone] or an inability to taper the dose to < 0.5 mg per kilogram per day of methylprednisolone or equivalent < 0.6 mg per kilogram per day of prednisone] for a minimum of 7 days). |
| Socie 2017, 2019 | GVHD progressing after ≥ 3 days of MP treatment, or GVHD persisting without improvement after 7 days of MP treatment. |
| Knop 2007 | NA |
| MacMillan 2007 | Continuing active GVHD despite treatment with methylprednisolone at doses of 2 mg/kg per day or higher or equivalent dose of another steroid for acute GVHD for at least 3 days, or failure during corticosteroid taper following initial treatment, which included 2 mg/kg per day or more of methylprednisolone or equivalent for at least 3 days. |
| Van Lint 2006 | Nonresponders to 6-methylprednisolone (6Mpred) at the dose of 2 mg/kg per day (failure to comply with the reduction of the dose of 6MPred on day +5; patients with acute GvHD progressing within day +5 were also eligible). |

Abbreviation: aGVHD, acute graft-versus-host disease; GVHD, graft-versus-host disease; MP, methylprednisolone; 6Mpred, 6-methylprednisolone; NA, not available; SR, steroid-refractory.
