# Supplementary material for: Second-line therapy for patients with steroid-refractory aGVHD: systematic review and meta-analysis of randomized controlled trials
Source: Front Immunol. 2023 Jun 20;14:1211171. doi: 10.3389/fimmu.2023.1211171 (PMC10318925; doi:10.3389/fimmu.2023.1211171)
Supplement: Supplementary file 6 [file Table_2.docx]

**Supplementary Table 2. Detailed dosage of second-line therapy regimens.**

| **Study** | **Treatment group** | **Dosage** |
| --- | --- | --- |
| Zhao 2022 (21) | MSC + Second-line therapy | MSCs (obtained from fresh BM of unrelated, HLA-mismatched, third-party donors) 1 × 10^6^ cells/kg given intravenously once weekly for 4 weeks as a cycle, PR patients continued to receive MSCs until aGVHD showed CR or MSCs had been infused for 8 doses; Second-line therapy given as the control group. |
|  | Second-line therapy | Basiliximab (20 mg per dose on day 1, 3, 8, and repeated weekly until aGVHD was reduced to grade < II) and calcineurin inhibitor (cyclosporine or tacrolimus) given in the first cycle. |
| Oosten 2022 (22) | MSC + Second-line therapy | MSCs 2 × 10^6^ cells/kg for 2 intravenous infusions at a 7-day interval; MMF as a standardized second-line therapy. |
|  | Second-line therapy | Placebo for 2 intravenous infusions; MMF as a standardized second-line therapy. |
| Kebriaei 2020 (23) | MSC + Second-line therapy | MSCs (Remestemcel-L, consisting of human culture-expanded MSCs from BM aspirates of unrelated, HLA-unmatched donors) 2 × 10^6^ cells/kg twice weekly for 4 weeks; Second-line therapy given as the control group. |
|  | Second-line therapy | Placebo at an equivalent volume; Second-line therapy according to a prespecified institutional standard of care (including ATG, MMF, infliximab, etanercept, daclizumab, pentostatin, denileukin diftitox, ECP, and alemtuzumab) |
| Zeiser 2020 (24) | Ruxolitinib | Ruxolitinib 10 mg twice daily given orally. Tapering of ruxolitinib was permitted after day 56 in patients who had a response. |
|  | Investigator’s choice | Investigator’s choice of therapy (including ATG, ECP, MSC, low-dose methotrexate, MMF, everolimus, sirolimus, etanercept, and infliximab) |
| Socie 2017, 2019 (25, 26) | Inolimomab | Inolimomab 0.3 mg/kg/day intravenously delivered for the induction phase, 0.2 mg/kg/day for maintenance. |
|  | ATG | ATG 2.5 mg/kg for 4 consecutive days. |
| Knop 2007 (27) | HD-MP + OKT3 | OKT3 5 mg intravenously once daily; MP 10 mg/kg, reduction of steroids was scheduled for the first 8 days and then subject to decision of the local investigator. |
|  | HD-MP | MP 10 mg/kg, reduction of steroids was scheduled for the first 8 days and then subject to decision of the local investigator. |
| MacMillan 2007 (28) | ABX-CBL | ABX-CBL 0.1 mg/kg/day intravenously administered for 14 days (induction regimen); Eligible patients received up to 3 maintenance courses (each course consisting of 2 infusions per week for 2 consecutive weeks.) |
|  | ATG | ATG 30 mg/kg/day every other day for a total of 6 doses; Eligible patients received additional courses. |
| Van Lint 2006 (29) | HD-MP + ATG | MP 5 mg/kg/day on days 6–15; ATG 1.25 mg/kg on days 6, 8, 10, 12, and 14. |
|  | HD-MP | MP 5 mg/kg/day on days 6–15. |

Abbreviation: ABX-CBL, a murine monoclonal antibody against CD147; aGVHD, acute graft-versus-host disease; ATG, anti-thymocyte globulin; BM, bone marrow; CR, complete response; ECP, extracorporeal photopheresis; HD-MP, high-dose methylprednisolone; HLA, human leukocyte antigen; MMF, mycophenolate mofetil; MP, methylprednisolone; MSC, mesenchymal stem cell; OKT3, a murine monoclonal antibody against CD3; PR, partial response.
